# Hyper, a Hydrogen Peroxide Sensor, Indicates the Sensitivity of the *Arabidopsis* Root Elongation Zone to Aluminum Treatment

**DOI:** 10.3390/s150100855

**Published:** 2015-01-06

**Authors:** Alejandra Hernández-Barrera, Ana Velarde-Buendía, Isaac Zepeda, Federico Sanchez, Carmen Quinto, Rosana Sánchez-Lopez, Alice Y. Cheung, Hen-Ming Wu, Luis Cardenas

**Affiliations:** 1 Department of Plant Molecular Biology, Institute of Biotechnology, National Autonomous University of Mexico, 62210 Mexico; E-Mails: alehb23@hotmail.com (A.H.-B.); anavela@ibt.unam.mx (A.V.-B.); z_isaac@hotmail.com (I.Z.); federico@ibt.unam.mx (F.S.); quinto@ibt.unam.mx (C.Q.); rosana@ibt.unam.mx (R.S.-L.); 2 Department of Biochemistry and Molecular Biology, University of Massachusetts, Amherst, MA 01003, USA; E-Mails: acheung@biochem.umass.edu (A.Y.C.); hmwu@biochem.umass.edu (H.-M.W.)

**Keywords:** Hyper, reactive oxygen species, aluminum, polar growth, *Arabidopsis*, plant root

## Abstract

Emerging evidence indicates that some reactive oxygen species (ROS), such as the superoxide anion radical and hydrogen peroxide (H_2_O_2_), are central regulators of plant responses to biotic and abiotic stresses. Thus, the cellular levels of ROS are thought to be tightly regulated by an efficient and elaborate pro- and antioxidant system that modulates the production and scavenging of ROS. Until recently, studies of ROS in plant cells have been limited to biochemical assays and the use of fluorescent probes; however, the irreversible oxidation of these fluorescent probes makes it impossible to visualize dynamic changes in ROS levels. In this work, we describe the use of Hyper, a recently developed live cell probe for H_2_O_2_ measurements in living cells, to monitor oxidative stress in *Arabidopsis* roots subjected to aluminum treatment. Hyper consists of a circularly permuted YFP (cpYFP) inserted into the regulatory domain of the *Escherichia coli* hydrogen peroxide-binding protein (OxyR), and is a H_2_O_2_-specific ratiometric, and therefore quantitative, probe that can be expressed in plant and animal cells. Now we demonstrate that H_2_O_2_ levels drop sharply in the elongation zone of roots treated with aluminum. This response could contribute to root growth arrest and provides evidence that H_2_O_2_ is involved in early Al sensing.

## 1. Introduction

In plant cells, ROS levels affect various processes, including development, the hypersensitive response to pathogen attack, hormone perception, gravitropism, and the stress response [1,2]. ROS regulate the opening of stomata in *Vicia faba* and have an important role in root hair and pollen tube growth in Arabidopsis. ROS levels generate and maintain the apical calcium gradient needed to support apical growth in root hair cells and pollen tubes [3–8].

ROS have been shown to function as key second messengers that regulate the conformation and activity of numerous proteins in animal cells [3,9]. Under physiological conditions, ROS production and removal should be tightly regulated to maintain optimal ROS levels. A ROS scavenging system, which involves several antioxidative defense mechanisms, eliminates excess ROS. However, increasing the activity of enzymes that generate ROS can perturb the balance between ROS production and removal and induce oxidative stress.

Whereas superoxide is neither membrane permeable nor long lasting, it can dismutate into hydrogen superoxide either spontaneously or by the activity of superoxide dismutase (SOD). Hydrogen superoxide gives rise to H_2_O_2_, which is considerably more stable than superoxide and is able to cross the plasma membrane, and thus functions as a long-range cell-to-cell signal [10]. It was recently reported that SOD is specifically relocated to the nucleus in response to H_2_O_2_, where it functions as a transcriptional factor [9,11].

Changes in ROS levels are recognized as specific signatures of plants in response to biotic and abiotic stresses [12–14]. Therefore, a change in ROS level at the cell wall induces a different response to that triggered by a similar change in ROS in the cytoplasm or in a given organelle. This is mainly due to the versatile role of ROS as second messengers, and their ability to induce specific responses. Reversible oxidation of proteins with adjacent cysteine residues, which might include protein kinases and phosphatases, can serve as an “on and off” switch that regulates protein function and redox signaling pathways in several stress responses [15,16].

Biochemical assays and fluorescent markers are widely used to analyze hydrogen peroxide production. For instance, 3,3′-diaminobenzidine (DAB) is a marker of H_2_O_2_ production [17]. Indeed, DAB has been used as frequently as Nitro Blue Tetrazolium (NBT) to image the superoxide oxidative burst during the plant-pathogen interaction, and several other biotic and abiotic responses [18]. However, both of these compounds are toxic and result in cell death. Therefore, DAB is useful for rapid in situ detection of H_2_O_2_ production, but not for dynamic analyses using living cells [17]. Fluorescein-derived compounds such as dihydrodichloroflorescein diacetate (H2DCF-DA) are widely used fluorescent probes for detecting H_2_O_2_ in living cells during various responses. However, H2DCF-DA is non-selective and thus several ROS can induce the unspecific oxidation of the probe. Furthermore, this probe is unstable in living cells, and is readily photooxidized and photobleached [18]. Finally, H2DCF-DA becomes irreversibly fluorescent once it reacts with ROS, and therefore a decline in ROS level will not be reported by this probe under normal conditions.

The newly developed molecular probe named Hyper consists of a circularly permuted YFP (cpYFP) molecule inserted into the regulatory domain of the *Escherichia coli* H_2_O_2_ sensor OxyR [19]. The activity of this regulatory domain relies on two cysteine residues, Cyst199 and Cys208. When the OxyR domain is exposed to H_2_O_2_, which promotes disulfide bond formation, an intramolecular change occurs shifting the excitation peak of the attached cpYFP from 420 to 500 nm, while the emission peak remains unchanged (at 516 nm) [19]. Due to this H_2_O_2_-induced change in excitation wavelength, Hyper can be used as a ratiometric biosensor. Because Hyper is reversibly modified, it is able to register both increases and decreases in H_2_O_2_ levels. Furthermore, since Hyper is a molecular probe, it can be introduced as a transgene and expressed in various organisms and targeted to specific cells and organelles, such as peroxisomes, mitochondria, and chloroplasts [20]. Thus, Hyper is a promising universal H_2_O_2_-specific biosensor for studies in plant cells [21].

Aluminium (Al) greatly limits plant growth and development in acidic soils, even when present at micromolar concentrations [22]. Although the mechanism by which Al inhibits root elongation is a topic of debate, there is some evidence that the primary lesions caused by Al toxicity are both apoplastic and symplastic, and that Al also affects the actin cytoskeleton [23–25]. Binding of Al to the pectic matrix of the cell wall and to the apoplastic face of the plasma membrane in the root apex, which is particularly sensitive to Al, can inhibit root elongation [24,26]. In addition, it has been reported that Al inhibits the activity of calcium channels and damages and peroxidates membrane lipids and the cytoskeleton [27]. As a result, root hair development is disrupted, the root apices swell up, and the roots stop growing [28]. However, the primary mechanism underlying Al toxicity is poorly understood, and a full characterization of Al-induced changes in plant cells is required to understand the toxic effect of Al [24].

Herein, we report the use of *Arabidopsis* lines expressing a Hyper transgene to detect the intracellular changes in H_2_O_2_ levels induced by Al. We found that plants exhibit a rapid decrease in intracellular ROS levels within the first minute of Al exposure. This decrease in ROS level is most pronounced in the elongation zone, where it induces permanent root growth arrest, but stimulates the generation of short secondary roots. This approach allows the visualization of ROS changes in living cells and provides an elegant strategy for studying stress responses in plants.

## 2. Experimental Section

*Arabidopsis thaliana* plants were transformed with *35s:HyPer* using the floral-dip method, as described [21,29]. Briefly, the dipped plants were allowed to grow until seed set. About 3 weeks after dipping, dried seeds were harvested and germinated on tissue culture medium (Gamborg B5, 1% sucrose, 5 mM MES pH 5.7, 0.7% agar, and 50 mg/L kanamycin) to screen for transformed plants. Kanamycin-resistant seedlings were transferred to soil and grown until flowering. The screening for transformants was carried out as follow: seeds were surface sterilized and germinated on tissue culture medium (Gamborg B5, 1% sucrose, 5 mM MES pH 5.7, and 0.7% agar) supplemented with 50 mg/L kanamycin. Kanamycin-resistant seedlings (T1 plants) emerged within two weeks amidst a lawn of dead (kanamycin-sensitive) seedlings. These T1 plants were transferred to soil, grown until flowering, and allowed to undergo self-fertilization to produce T2 seeds. These T2 seeds were then germinated on medium consisting of Gamborg B5, 1% sucrose, 0.7% agar. Roots from these T2 seedlings (∼1 week old) were examined for YFG/GFP fluorescence and for sensitivity to a H_2_O_2_-induced shift in excitation maximum, and seedlings with a good signal were selected, while those with very high and low levels of signal were discarded. Some of the YFP-positive seedlings were transferred to soil again and grown to maturity for seed collection. These T3 seeds and seeds from future generations were used in the experiments.

### 2.1. Seed Sterilization and Stratification

Arabidopsis seeds were surface sterilized using the vapor phase method [29]. The sterilized seeds were re-suspended in 1 ml of sterilized water and stratified by incubation at 4 °C for 2 days, in the dark, to improve the rate and synchrony of germination.

### 2.2. Growth Conditions

The plants were grown in Linsmaier and Skoog basal medium (L477, Phyto Technology Laboratories, Lenexa, KS, USA) that had been supplemented with Linsmaier and Skoog vitamins (thiamine·HCl and myo-inositol), adjusted to pH 5.25–6.25, and buffered with MES. Briefly, surface-sterilized seeds were inserted to a depth of around 5–7 mm in the solid medium cast in modified Petri dishes containing coverslips in their centers for better microscopy observation [21]. The dishes were closed and sealed with Parafilm and placed horizontally in a growth chamber set to deliver 16 h light (105 μmol photons m^−2^·s^−1^) and 8 h darkness at 21 °C. After 3 days of germination, when the primary roots had reached the coverslip at the bottom of the chamber, the Petri dishes were positioned vertically to promote root growth over the surface of the coverslip and thereby permit fluorescence microscopy observations. Plant roots were treated with several concentration of AlCl_3_ by making a hole in the agar medium close to the root and filling the hole with Al solution. Usually the solution perfused to the root in less than a minute. For the long-term (5-day) experiments, we supplemented the entire solid medium with Al and recorded our observations after 4–5 days; in order to avoid evaporation the plates were sealed.

### 2.3. Image Acquisition and Processing

All images were acquired with a CCD camera (Sensys, Roper Scientific, Tucson, AZ, USA) attached to a TE300 inverted microscope (Nikon, Japan) coupled to a xenon illumination source (DG-4, Sutter Instruments, Novato, CA, USA), which contained a 175-Watt ozone-free xenon lamp (330–700 nm) and a galvanometer-driven wavelength switcher. A Uniblitz shutter (Vincent Associates, Rochester, NY, USA), which allows the acquisition of the transmitted light for every ratio image, was incorporated. Briefly, to make a ratiometric analysis, a filter wheel was used to switch between two different excitation wavelengths, 440 nm for the H_2_O_2_-independent signal and 495 nm for the H_2_O_2_-dependent signal, and all the emission spectra were collected using an emission filter at 530/20 nm. An extra image corresponding to the transmitted light was acquired to register morphological changes and changes in growth. Images were acquired with a 20X water immersion lens (Nikon; N.A. 0.9) with a 2 mm working distance. All of these systems were operated by MetaMorph/MetaFluor software (Universal Imaging-Molecular Devices, Downingtown, PA, USA).

## 3. Results and Discussion

**Figure 1.**
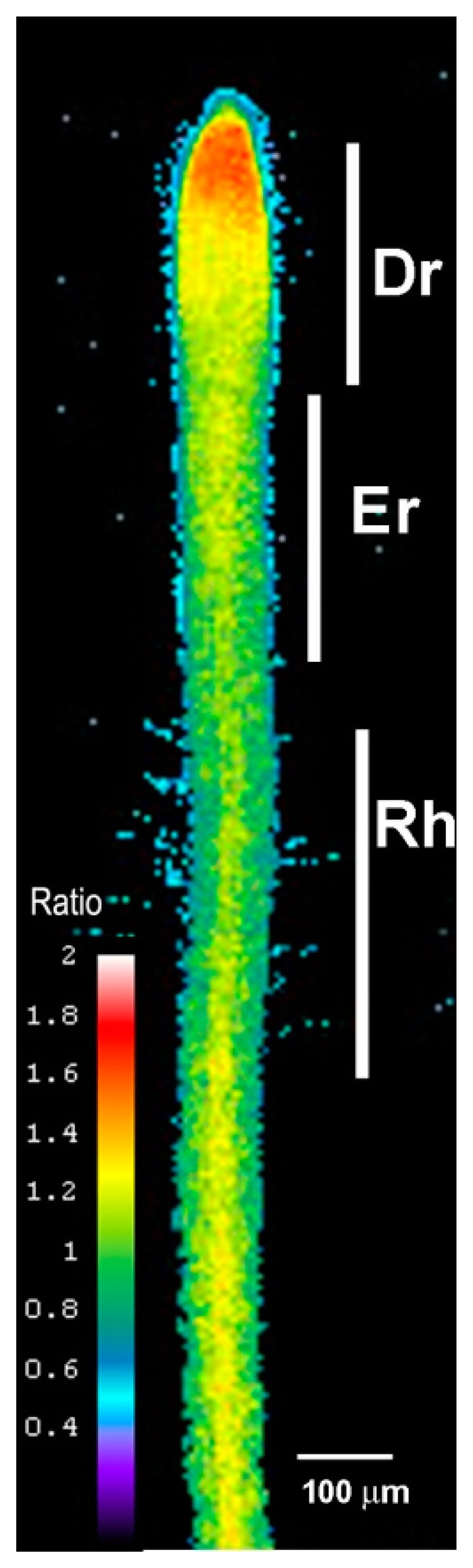
Hyper expression in transgenic Arabidopsis lines depicts the H_2_O_2_ distribution in the growing root. Note the high H_2_O_2_ levels in the root tip region where cell division occurs (Dr) and the lower levels in the elongation zone (Er) and root hairs at the root hair emerging zone (Rh).

We examined the roots of 5-day-old transgenic *Arabidopsis* seedlings expressing Hyper using ratiometric fluorescence imaging. Hyper depicts a clear H_2_O_2_ distribution that is abundant in the root tip region, labeled the division region (Dr), and is present at lower amounts in the elongation region (Er), including the region of root hair cell formation (Rh). The distribution shown in Figure 1 is representative of that in a normal root growing at a normal rate (*i.e.*, half a centimeter a day under our growth conditions).

**Figure 2.**
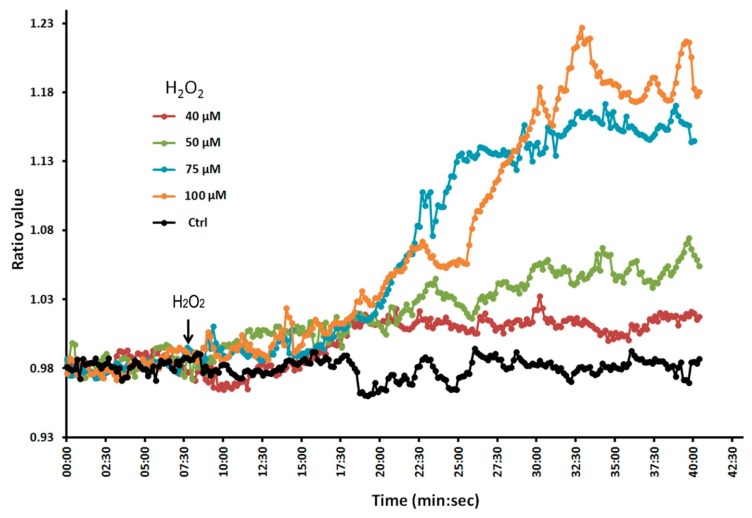
Increased levels of external H_2_O_2_ result in increased intracellular changes in ratio values. Arabidopsis roots expressing Hyper were treated with different external concentrations of H_2_O_2_ and the intracellular changes were monitored by analyzing Hyper fluorescence ratios. Images were acquired at 10 s intervals. Note that higher concentrations of external H_2_O_2_ resulted in increased ratio values.

To confirm that the Hyper lines do indeed respond to H_2_O_2_, we conducted a dose-response experiment in which we treated the plant roots with different concentrations of H_2_O_2_ (Figure 2). We found that fluorescence ratio values increased with increasing concentrations of H_2_O_2_, indicating that the molecular probe is indeed able to respond to changes in H_2_O_2_. We monitored the plant roots for up to 4 h without observing any photobleaching effects. Thus, these Hyper lines are suitable for evaluating the effect of Al on intracellular H_2_O_2_ levels in *Arabidopsis* roots in experiments lasting from 1–2 h.

### 3.1. H_2_O_2_ Rapidly Dissipates in the Elongation Zone of Roots Treated with Al

**Figure 3.**
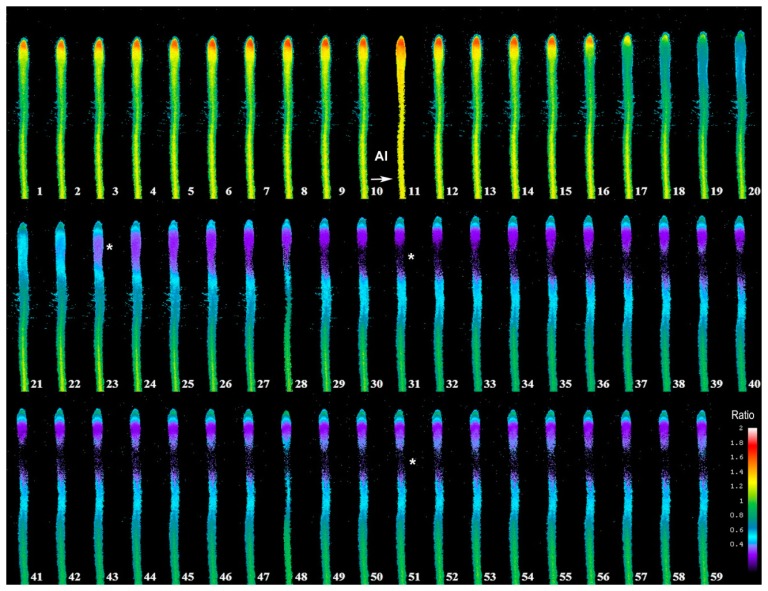
Exposure to Al (800 nM) induces dramatic changes in intracellular H_2_O_2_ levels in growing *Arabidopsis* roots. Note that H_2_O_2_ levels are highest in the root tip, but that the elongation zone exhibits the strongest response to Al treatment. Furthermore, the differentiation zone, from where root hairs emerge, is also affected. The arrow indicates when the Al was added and the asterisks where the elongation zone is located. Images were acquired at 1-min intervals.

**Figure 4.**
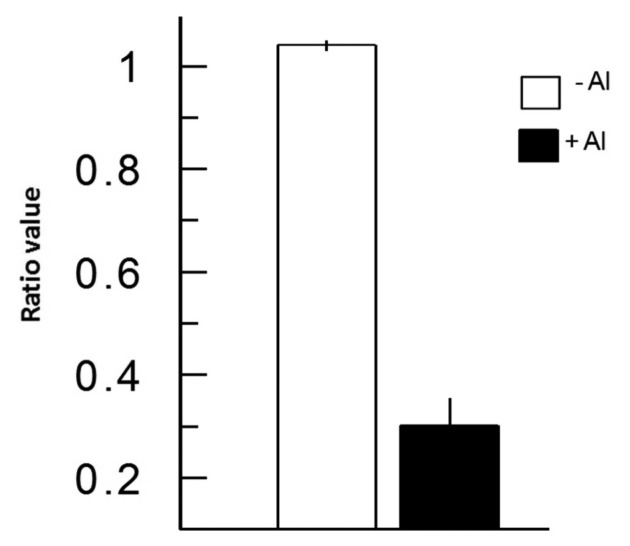
The elongation region depicts dramatic decreases in intracellular H_2_O_2_ levels. Intracellular H_2_O_2_ levels were measured in several *Arabidopsis* roots before and after treatment with 1 mM Al. Note that the elongation region exhibits a dramatic decrease in ratio signal after 30 min of Al treatment. *n* = 25 cells.

**Figure 5.**
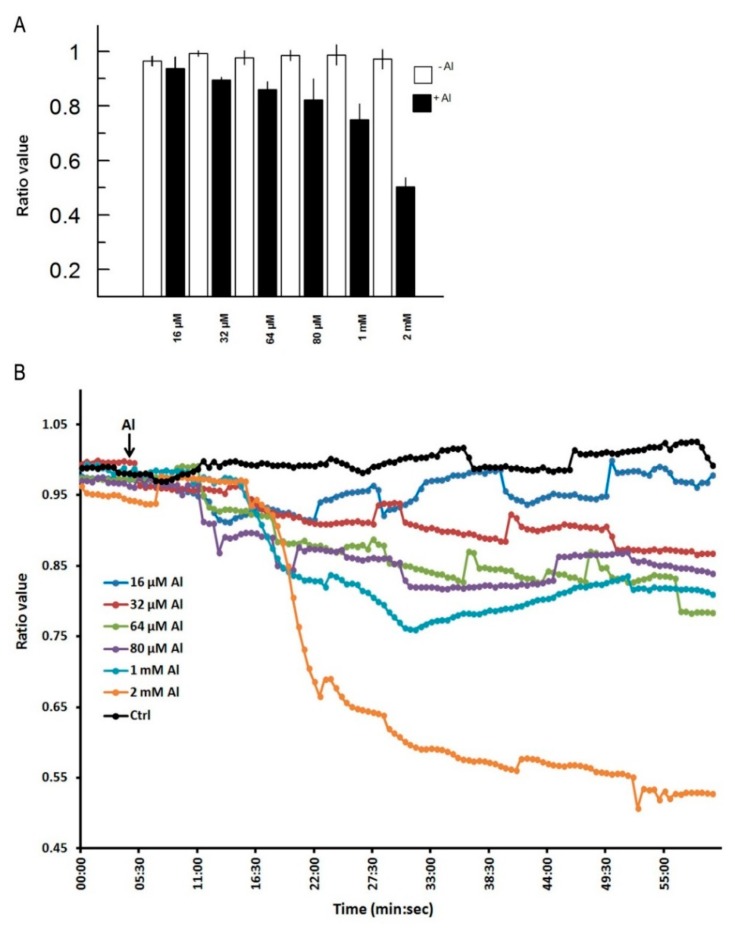
Intracellular levels of H_2_O_2_ in *Arabidopsis* roots respond to Al treatment in a dose-dependent manner. (**A**) Roots were treated with different Al concentrations and the intracellular H_2_O_2_ levels were evaluated as a ratio change of the Hyper probe. Note that higher concentrations of Al induce lower intracellular H_2_O_2_ levels. *n* = 25 cells; (**B**) Graph depicting the representative response of *Arabidopsis* roots to various Al concentrations. Images were acquired at 30 s intervals.

In Figure 3, we depict a series of images from the same root responding to Al. Within 5 min of Al treatment, the roots exhibited a general decrease in intracellular H_2_O_2_ concentration; however, the most dramatic decrease occurred in the elongation region (see asterisk), in which the signal vanished. The H_2_O_2_ concentration in the root hair differentiation zone also declined sharply. After 15 min, root growth was arrested and did not reinitiate, even after several hours. It is well known that the elongation zone is the region where roots elongate at a rapid rate, and it has been suggested that Al targets these cells [30]. To determine the sensitivity of the elongation zone to Al treatment, we measured H_2_O_2_ levels before and after Al treatment (Figure 4). The fluorescence ratio values were around 1 before the treatment, but decreased to 0.3 after the treatment. This response is dose dependent, since treatment with different concentrations of Al resulted in different degrees of response (Figure 5, panel A). In Figure 5 (panel B), we depict a representative experiment showing the response to treatment with various Al concentrations in the elongation zone. Note that higher concentrations of Al were associated with lower fluorescence ratio values. Again, there is a strong correlation between the Al concentration and intracellular levels of H_2_O_2_. We then measured the intracellular H_2_O_2_ levels in different regions of the root after Al treatment.

**Figure 6.**
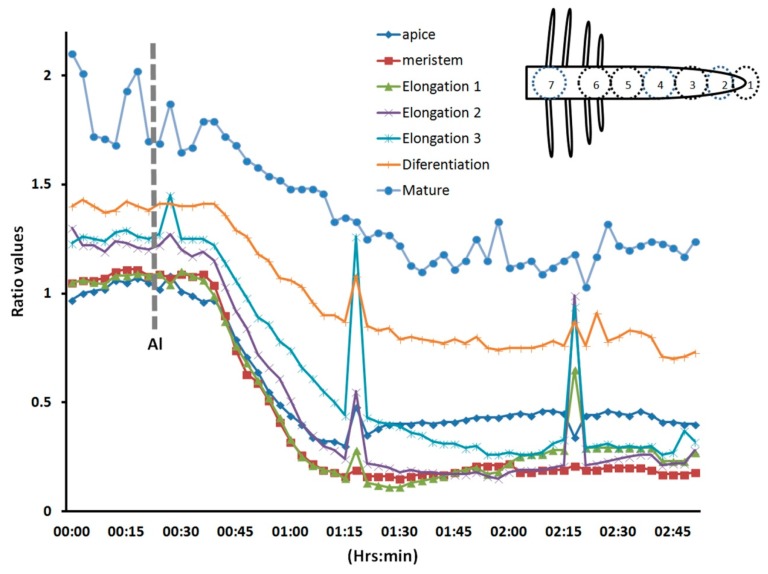
Different regions of the roots respond with a similar pattern, but the elongation region exhibits the most dramatic change. Different regions of *Arabidopsis* roots treated with 1 mM Al were evaluated for changes in intracellular H_2_O_2_ levels (see inset). Note that all the regions responded at the same time, but with a different strength. 1 = apical, 2 = meristem, 3 = elongation 1, 4 = elongation 2, 5 = elongation 3, 6 = differentiation, 7 = mature.

We selected the root cap, the meristem region, three sub-regions (1, 2, and 3) of the elongation zone, the differentiating region (where the root hairs emerge), and the mature region (where the root hairs have ceased growing) (Figure 6). In this experiment, we observed that all regions responded to Al treatment; however, the elongation zone seemed to be more sensitive, and the mature region more resistant to Al. These results were consistently observed in all roots analyzed (*n* = 10). Some time we observed some abrupt rises in the intracellular H_2_O_2_, however they were lower than the initial values. It could be an intention to reestablish the original H_2_O_2_ distribution; however the general response is a decreased intracellular level. Our results suggest that H_2_O_2_, an important regulator of plant cell growth [2], is more readily dissipated in cells of the elongation zone than in other regions of the root upon Al treatment. This early Al-induced decrease in intracellular H_2_O_2_ has not been described before in living plant roots, and thus is the first time that a biosensor has been used to depict this cellular response. The decreased H_2_O_2_ distribution could be due to the effect of Al on the cell wall. Al stress is known to increase the amounts of certain cell wall components, such as polysaccharides, hemicelluloses, extensins, callose, and lignin [26]. These changes in cell wall composition could have a negative impact on H_2_O_2_-producing enzymes, such as apoplastic diamine oxidase or NADPH oxidases, and even when these enzymes produce superoxide, these molecules can be dismutated into H_2_O_2_. On the other hand, the epidermal cells are very susceptible to Al accumulation, and Al likely promotes epidermal cell death, resulting in growth inhibition [26]. In addition, Al binding to the plasma membrane could have a strong effect on the cytoskeleton and signal transduction pathways, which could in turn cause growth inhibition [25]. It has recently been suggested that receptor-like kinases that have a malectin domain, such as Feronia, Anxur, and Theseus, are key components of cellular surveillance mechanisms that monitor the cell wall. Furthermore, these receptors are important modulators of NADPH oxidases [12,31,32]. This could explain the rapid decrease in intracellular H_2_O_2_ levels after Al treatment.

#### 3.1.1. Intracellular H_2_O_2_ Levels Increase in the Roots of Plants Subjected to Prolonged Al Treatment

**Figure 7.**
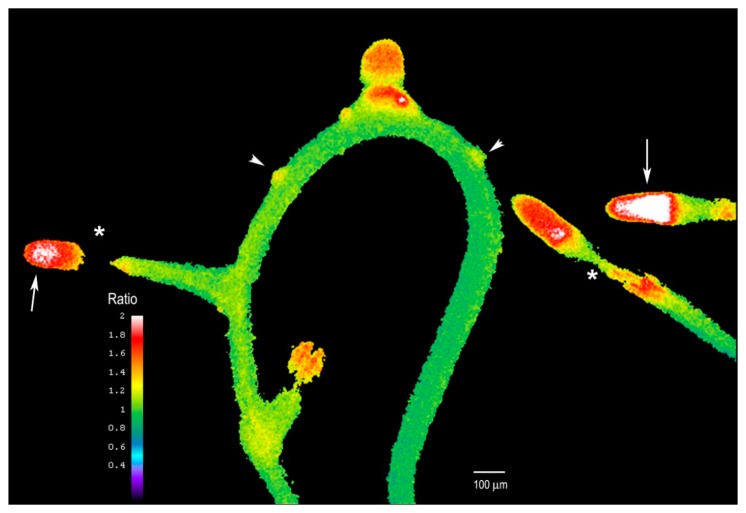
*Arabidopsis* roots exhibit increased intracellular H_2_O_2_ levels after several days of exposure to Al. After 5 days of Al treatment, roots responded with an increase in lateral root formation. The newly formed lateral roots were shorter than those of control plants of the same age and had higher H_2_O_2_ levels in the tip regions. However, the elongation zone remained and exhibited reduced H_2_O_2_ levels. Arrows indicate the tip of the root where the division region is located, asterisks the elongation zone, and arrowheads the small lateral root primordia.

We next examined whether the plant's response to Al differed after extended Al treatment where we supplemented the entire solid medium with Al and sealed the Petri dish to avoid evaporation. After five days of treatment, the growth of the main root remains arrested, but many new lateral roots had formed (see Figure 7). These roots were short and exhibited higher intracellular H_2_O_2_ levels, especially in the tip region (arrows, Figure 7) as compared to the rest of the root. However, the H_2_O_2_ levels in the elongation zone of these lateral roots were particularly suppressed, and this region exhibited the lowest levels (asterisks, Figure 7).

While cessation of root growth followed by the formation of lateral roots upon Al treatment was previously described [30], a connection has not been made between Al treatment and intracellular H_2_O_2_ dynamics. These data suggest that H_2_O_2_ levels in the root are tightly regulated to support optimal root growth, or that a sustained increase in H_2_O_2_ could affect the meristematic region of the main root, inducing the formation of many lateral roots to compensate for the effect of Al. In fact, it has been reported that it could take up to 24 h for Al to reach the internal cells of the root, including the meristems, and this could explain why after several days we see the lateral root induction and higher H_2_O_2_ levels. It is clear that numerous lateral root primordia form after extended Al treatment (arrowheads, Figure 7), probably due to Al accumulation in internal tissues, but they still fail to elongate fully, suggesting that Al could affect the root growth and cell elongation. Thus, we found Hyper to be an excellent biosensor for monitoring H_2_O_2_ levels even after several days of Al treatment. We propose that this probe could be used to explore how H_2_O_2_ is involved in other biological processes or stress responses.

## 4. Conclusions/Outlook

*Arabidopsis* plants expressing Hyper, a biosensor for intracellular H_2_O_2_, represent a powerful tool for visualizing oxidative stress under Al treatment. We conducted a dose response analysis to demonstrate that the ratio values of the probe respond with a clear increase to increasing concentrations of H_2_O_2_. This approach allowed us to characterize the effect of Al on the *Arabidopsis* root and determine that the elongation zone of the root is the most susceptible region and responded in a dose-dependent manner to several Al concentrations. Since intracellular ROS levels affect plant cell growth, it is reasonable to propose that the drastic decrease in intracellular H_2_O_2_ in the elongation zone after Al treatment affects root growth. On the other hand, this growth inhibition explains the stunted phenotype and poor growth under Al stress. We do not discard the possibility that other factors upstream of H_2_O_2_ are involved in root growth inhibition in response to Al treatment. For instance, calcium and pH changes may well be involved in the same pathway. It is possible that the concentration of other free radicals, such as superoxide or hydroxyls, could increase rather than decrease in response to Al treatment. Indeed, calcium is an important agonist in the activation of NADPH oxidase, a ROS generating enzyme. All of these factors could directly or indirectly result in cell wall rigidification and growth arrest. Finally, we show that the Hyper biosensor can be used to visualize H_2_O_2_ dynamics in all parts of a plant by fluorescence microscopy. Thus, this approach can be used in studies that evaluate plant responses at the cellular level, and can differentiate between responses in the root tip, root hair, elongation zone, and vascular bundles of primary and secondary roots.

## References

[1] Mittler R., Berkowitz G. (2001). Hydrogen peroxide, a messenger with too many roles?. Redox Rep..

[2] Tsukagoshi H., Busch W., Benfey P.N. (2010). Transcriptional regulation of ROS controls transition from proliferation to differentiation in the root. Cell.

[3] Mori I.C., Schroeder J.I. (2004). Reactive oxygen species activation of plant Ca^2+^ channels. A signaling mechanism in polar growth, hormone transduction, stress signaling, and hypothetically mechanotransduction. Plant Physiol..

[4] Foreman J., Demidchik V., Bothwell J.H., Mylona P., Miedema H., Torres M.A., Linstead P., Costa S., Brownlee C., Jones J.D. (2003). Reactive oxygen species produced by NADPH oxidase regulate plant cell growth. Nature.

[5] Cárdenas L., Quinto C. (2008). Reactive oxygen species (ROS) as early signals in root hair cells responding to rhizobial nodulation factors. Plant Signal. Behav..

[6] Pei Z.M., Murata Y., Benning G., Thomine S., Klusener B., Allen G.J., Grill E., Schroeder J.I. (2000). Calcium channels activated by hydrogen peroxide mediate abscisic acid signalling in guard cells. Nature.

[7] Kaya H., Nakajima R., Iwano M., Kanaoka M.M., Kimura S., Takeda S., Kawarazaki T., Senzaki E., Hamamura Y., Higashiyama T. (2014). Ca^2+^-activated reactive oxygen species production by Arabidopsis RbohH and RbohJ is essential for proper pollen tube tip growth. Plant Cell.

[8] Lassig R., Gutermuth T., Bey T.D., Konrad K.R., Romeis T. (2014). Pollen tube NAD(P)H oxidases act as a speed control to dampen growth rate oscillations during polarized cell growth. Plant J..

[9] Marinho H.S., Real C., Cyrne L., Soares H., Antunes F. (2014). Hydrogen peroxide sensing, signaling and regulation of transcription factors. Redox Biol..

[10] Allan A.C., Fluhr R. (1997). Two Distinct Sources of Elicited Reactive Oxygen Species in Tobacco Epidermal Cells. Plant Cell.

[11] Tsang C.K., Liu Y., Thomas J., Zhang Y., Zheng X.F. (2014). Superoxide dismutase 1 acts as a nuclear transcription factor to regulate oxidative stress resistance. Nat. Commun..

[12] Wolf S., Hofte H. (2014). Growth Control: A Saga of Cell Walls, ROS, and Peptide Receptors. Plant Cell.

[13] Torres M.A. (2009). ROS in biotic interactions. Physiol. Plant.

[14] Swanson S., Gilroy S. (2009). ROS in plant development. Physiol. Plant.

[15] Corcoran A., Cotter T.G. (2013). Redox regulation of protein kinases. Febs. J..

[16] Moran L.K., Gutteridge J.M., Quinlan G.J. (2001). Thiols in cellular redox signalling and control. Curr. Med. Chem..

[17] Driever S.M., Fryer M.J., Mullineaux P.M., Baker N.R. (2009). Imaging of reactive oxygen species in vivo. Methods Mol. Biol..

[18] Swanson S.J., Choi W.G., Chanoca A., Gilroy S. (2011). *In vivo* imaging of Ca^2+^, pH, and reactive oxygen species using fluorescent probes in plants. Annu. Rev. Plant Biol..

[19] Belousov V.V., Fradkov A.F., Lukyanov K.A., Staroverov D.B., Shakhbazov K.S., Terskikh A.V., Lukyanov S. (2006). Genetically encoded fluorescent indicator for intracellular hydrogen peroxide. Nat. Methods..

[20] Costa A., Drago I., Behera S., Zottini M., Pizzo P., Schroeder J.I., Pozzan T., Lo Schiavo F. (2010). H_2_O_2_ in plant peroxisomes: An *in vivo* analysis uncovers a Ca(^2+^)-dependent scavenging system. Plant J..

[21] Hernández-Barrera A., Quinto C., Johnson E.A., Wu H.M., Cheung A.Y., Cardenas L. (2013). Using hyper as a molecular probe to visualize hydrogen peroxide in living plant cells: A method with virtually unlimited potential in plant biology. Methods Enzymol..

[22] Kochian L.V., Hoekenga O.A., Pineros M.A. (2004). How do crop plants tolerate acid soils? Mechanisms of aluminum tolerance and phosphorous efficiency. Annu. Rev. Plant Biol..

[23] Amenos M., Corrales I., Poschenrieder C., Illes P., Baluska F., Barcelo J. (2009). Different effects of aluminum on the actin cytoskeleton and brefeldin A-sensitive vesicle recycling in root apex cells of two maize varieties differing in root elongation rate and aluminum tolerance. Plant Cell Physiol..

[24] Horst W.J., Wang Y., Eticha D. (2010). The role of the root apoplast in aluminium-induced inhibition of root elongation and in aluminium resistance of plants: A review. Ann. Bot..

[25] Blancaflor E.B., Jones D.L., Gilroy S. (1998). Alterations in the cytoskeleton accompany aluminum-induced growth inhibition and morphological changes in primary roots of maize. Plant Physiol..

[26] Jones D.L., Blancaflor E.B., Kochian L.V., Gilroy S. (2006). Spatial coordination of aluminium uptake, production of reactive oxygen species, callose production and wall rigidification in maize roots. Plant Cell Environ..

[27] Jones D.L., Kochian L.V. (1995). Aluminum Inhibition of the Inositol 1,4,5-Trisphosphate Signal Transduction Pathway in Wheat Roots: A Role in Aluminum Toxicity?. Plant Cell.

[28] Panda S.K., Baluska F., Matsumoto H. (2009). Aluminum stress signaling in plants. Plant Signal Behav..

[29] Clough S.J., Bent A.F. (1998). Floral dip: A simplified method for Agrobacterium-mediated transformation of Arabidopsis thaliana. Plant J..

[30] Ma J.F., Shen R., Nagao S., Tanimoto E. (2004). Aluminum targets elongating cells by reducing cell wall extensibility in wheat roots. Plant Cell Physiol..

[31] Boisson-Dernier A., Lituiev D.S., Nestorova A., Franck C.M., Thirugnanarajah S., Grossniklaus U. (2013). ANXUR receptor-like kinases coordinate cell wall integrity with growth at the pollen tube tip via NADPH oxidases. PLoS Biol..

[32] Duan Q., Kita D., Li C., Cheung A.Y., Wu H.M. (2010). FERONIA receptor-like kinase regulates RHO GTPase signaling of root hair development. Proc. Natl. Acad. Sci. USA.

